# Congenital hypoplasia of first digital ray of hands as an isolated presentation in four subjects

**DOI:** 10.12669/pjms.306.5464

**Published:** 2014

**Authors:** Karmoon Lal, Sara Mumtaz, Attiq-ur- Rehman, Maryam Bibi, Zahida Pervin, Sajid Malik

**Affiliations:** 1 Karmoon Lal, Human Genetics Program, Department of Animal Sciences, Faculty of Biological Sciences, Quaid-i-Azam University, 45320 Islamabad, Pakistan.; 2 Sara Mumtaz, Human Genetics Program, Department of Animal Sciences, Faculty of Biological Sciences, Quaid-i-Azam University, 45320 Islamabad, Pakistan.; 3 Attiq-ur-Rehman, Human Genetics Program, Department of Animal Sciences, Faculty of Biological Sciences, Quaid-i-Azam University, 45320 Islamabad, Pakistan.; 4 Maryam Bibi, Human Genetics Program, Department of Animal Sciences, Faculty of Biological Sciences, Quaid-i-Azam University, 45320 Islamabad, Pakistan.; 5 Zahida Pervin, Human Genetics Program, Department of Animal Sciences, Faculty of Biological Sciences, Quaid-i-Azam University, 45320 Islamabad, Pakistan.; 6 Sajid Malik, Human Genetics Program, Department of Animal Sciences, Faculty of Biological Sciences, Quaid-i-Azam University, 45320 Islamabad, Pakistan.

**Keywords:** Thumb hypoplasia, First digit ray, Oligodactyly, Limb anomaly, Pakistani subjects

## Abstract

Congenital hypoplasia of thumb is rare malformation which is less likely to appear as an isolated entity. Four independent subjects exhibiting various grades of underdeveloped first digital ray were recruited. The affected autopods had narrow palms, medial or valgus inclinations of index fingers and thenar weakness, while the postaxial digits were least affected. According to the classification of hypoplastic thumb by Blauth and Schneider-Sickert (1981), the phenotypes were concordant with types 3 and 4. In one of the subjects there was contralateral preaxial polydactyly. All cases were sporadic and nonsyndromic and parental consanguinity was witnessed in two individuals. Recurrent appearance of similar phenotypes may suggest genetic etiologies which should be elucidated with the help of high-throughput genetic methods.

## INTRODUCTION

Congenital hypoplasia of first digital ray (OMIM-188100) is a rare situation of underdeveloped thumb and is characterized by varying degrees of incompletely developed first digital ray, contracted first web space, unstable metacarpophalangeal joint and thenar weakness.^1^^,^^2^ Thumb hypoplasia has been reported to comprise 3.5% of congenital upper-limb malformations.^3^ This anomaly is witnessed less often as an isolated entity while syndromic occurrences are frequent, and the associations with radial deficiency and musculoskeletal, craniofacial, cardiac, renal, gastrointestinal, and hematopoietic symptoms have been observed.^1^^,^^4^ In Pakistan, there are only few studies available on limb deficiencies.^5^^-^^7^ Here, we report on four independent subjects presented with isolated and sporadic thumb hypoplasias.

## SUBJECTS

**Fig.1A:**
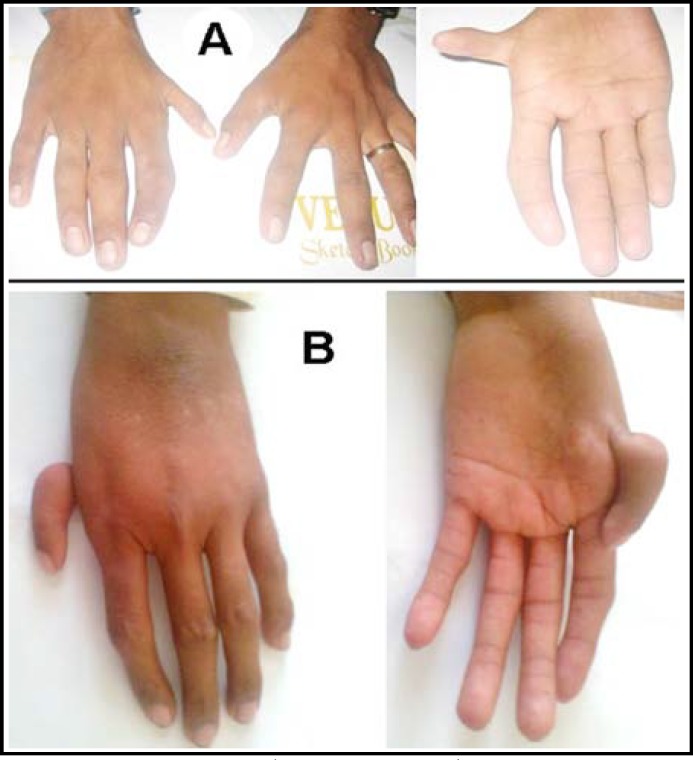
Phenotype in Subject I.

Four subjects (3 Males, 1 Female) with underdeveloped first digital ray of hands were ascertained during Mar. 2010-Nov. 2013 (Table-I). The cardinal presentation in all subjects was thumb hypoplasia/apalsaia which was characterized with the schemes of Blauth and Schneider-Sickert^8^ and James et al.^9^ There was occasional involvement of other fingers, but there was no involvement of lower-limbs or other organ-systems. Duplication of thumb observed in one subject was classified according to Malik.^10^ A snapshot of phenotypic presentation in each subject is given below (Table-II):

***Subject I: ***This male subject, 15 years, belonged to interior Balochistan. His right thumb was hypoplastic and proximally set. There was medial inclination of the right index finger at the proximal phalangeal joint (Fig.1A). The left hand was unremarkable.

***Subject II: ***This male subject had unilateral hypoplastic thumb in the left hand (Fig.1B). The left thumb was replaced by a dwarf digit which was passively hanging through a rudimentary skin tag. It contained bony elements and a hypoplastic nail. The fingers 2-5 were long and cylindrical and exhibited extra flexion creases.

**Fig.2A:**
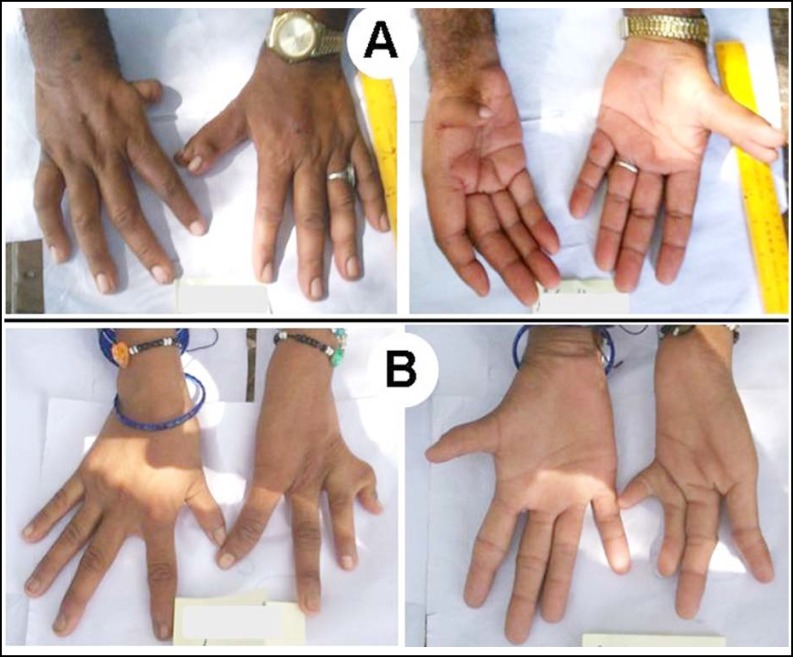
Phenotype in Subject III.

***Subject III: ***This male subject belonged to interior Sindh. He was observed to have hypoplastic right thumb which was low-set and proximally placed (Fig.2A). Its attachment with the autopod was feeble and it showed contracture at the interphalageal joint. Additionally, the left thumb was bifid at the proximal phalange and harbored duplicated distal phalanx with separate nails. The subject was the product of a consanguineous union and had fathered three unaffected offspring.

***Subject IV: ***This female subject demonstrated hypoplasia of right thumb with weak thener muscles (Fig.2B). In the left hand, there was complete absence of thumb, radial inclination of index finger, and brachysyndactyly of 4^th^-5^th^ fingers. Particularly, the 5^th^ digit was considerably short and demonstrated radial splaying.

## DISCUSSION

The revised classification of hypoplastic thumb by Blauth and Schneider-Sickert^8^, and James et al.^9^ is based on the anatomical scheme and is also useful from the viewpoint of treatment considerations. This scheme identifies five entities of increasing severity. Among the four affected thumbs in the recruited subjects, a rather severe phenotype was witnessed which was concordant with types 3B and 4 according to the Blauth/Schneider-Sickert scheme, while in subject IV left thumb was completely omitted (consistent with type 5). The common manifestations in the observed affected limbs/autopods were: thumb hypoplasia (n=4), deviations (medial or valgus) of index fingers (n=3), campto-/brachy-dactyly of 5^th^ digits (n=2), short and narrow palm (n=2), reduced arm length (n=2), and thumb agenesis (n=1). Affected autopods had remarkable dermatoglyphics. In subject III, there was thumb hypoplasia and contralateral bifid thumb. Association of thumb hypoplasia/aplasia with polydactyly has not been much elucidated and is an interesting question for further studies. In the current study, another common finding with thumb anomaly was a short arm. However, we did not observe bowing of zeugopod (a severe consequence of radial dysplasia), as is usually reported with thumb hypoplasia/aplasia.^4^

The prevalence of congenital limb anomalies has been proposed to be higher in subjects with primi-gravida and with high parental consanguinity. In the present cohort, none of them belonged to primi-gravida and parental consanguinity was observed in only two of the cases. In Pakistan, there is no study available showing the prevalence of congenital limb deficiencies. Research is also scarce regarding the socio-demographic attributes and risk factors for the isolated types of limb deficiencies. Furthermore, phenotypic manifestations of limb deficiencies and associations with dysmorphologies of other organ-systems have not been much explored in Pakistan mainly due to the rarity of these anomalies.^5^ Hence, the burden of such disorders on our population remains to be elucidated. 

**Table-I:** Socio-demographic and biological attributes of recruited subjects.

**Variable**	**Subjects**			
	**I**	**II**	**III**	**IV**
Gender (M,F)	M	M	M	F
Age (year)	15	42	40	12
Origin	Interior Balochistan	Punjab	Interior Sindh	Interior Sindh
Rural/Urban	U	U	R	U
Caste	Baloch	Niazi	Muslim/Machi	Hindu/Odd
Language/ethnicity	Balochi	Saraiki	Sindhi	Dhatki
Socio-economics	Poor	Low	Poor	Poor
Family/house-hold type	Nuclear	Nuclear	Nuclear	Nuclear
Education/occupation	Nil/manual jobs	Primary/ manual jobs	Primary/manual jobs	Student
Marital status	Single	Married	Married	Single
Parental Consanguinity	UR	FC	FC	UR
Father age at subject’s birth (year)	25	32	45	28
Mother age at subject’s birth (year)	20	29	35	25
Subject’s parity	2-of-6	2-of-3	3-of-3	4-of-6
Normal sibs (Brother:Sister)	1:4	1:1	2:0	3:2

FC=first cousins; UR=unrelated.

**Table-II:** Phenotypic manifestations of thumb hypoplasia in recruited subjects.

**Phenotype**	**Subjects**			
	**I**	**II**	**III**	**IV**
Right thumb	Hypoplastic	Unaffected	Hypoplastic	Hypoplastic
Left thumb	Unaffected	Hypoplastic/floating	Bifid*	Absent
Index finger, medial inclination	R++	L+		L. valgus deviation
Other digits		Thin/cylindrical fingers	R. camptodactyly of 5^th^	L. brachy-syndactyly of 4-5
Palm, thin/reduced		L+		L+
Arm, reduced/short		L+		L+
Classification	Type-3B	Type-4	Type-4	Type-3B in R; type-5 in L

L=left; R=right;

+,++ =severity grades

* preaxial polydactyly type-I

1B: Dorsal and ventral aspect of left hand of Subject II.

2B: Hands of Subject IV**.**

Thumb is a remarkable finger with unique anatomical and functional characteristics. Underdeveloped thumb is often detrimental to hand function. Hence, the anomalies of thumb deserve a separate attention among the hand defects.^1^ In Pakistan, majority of the subjects with certain types of hand defects are unable to gain attention of surgeons. A large number of subjects remain untreated mainly due to unavailability of specialized doctors and the high cost of surgical procedures. It is need of the hour to initiate a rehabilitation program in the country for the functional and aesthetic reconstruction for subjects with major and minor types of congenital limb defects.

## Notes

***Financial Assistance: ***This study was supported by the HEC-Islamabad, and PSF-Pakistan.
